# Designing Artificial Environments for Preterm Infants Based on Circadian Studies on Pregnant Uterus

**DOI:** 10.3389/fendo.2013.00113

**Published:** 2013-09-04

**Authors:** Shimpei Watanabe, Shizuko Akiyama, Takushi Hanita, Heng Li, Machiko Nakagawa, Yousuke Kaneshi, Hidenobu Ohta, Japan RED filter study group

**Affiliations:** ^1^Department of Neonatology, Kanagawa Children’s Medical Center, Yokohama, Japan; ^2^Center for Perinatal Medicine, Tohoku University Hospital, Sendai, Japan; ^3^Department of Pediatrics, Tohoku University Hospital, Sendai, Japan; ^4^Department of Anatomy and Developmental Biology, School of Biomedical Sciences, Faculty of Medicine, Nursing and Health Sciences, Monash University, Clayton, VIC, Australia; ^5^Department of Developmental Disorders, National Institute of Mental Health, National Center of Neurology and Psychiatry, Kodaira, Tokyo, Japan; ^6^Department of Pediatrics, St Luke’s International Hospital, Chuo-ku, Tokyo, Japan; ^7^Center for Perinatal Medicine, Hokkaido University Hospital, Sapporo, Japan

**Keywords:** designing artificial environments, preterm infants, circadian clocks, pregnant uterus, lighting conditions

## Abstract

Using uterine explants from Per1::Luc rats and *in situ* hybridization, we recently reported that the circadian property of the molecular clock in the uterus and placenta is stably maintained from non-pregnancy, right through to the end stage of pregnancy under regular light-dark (LD) cycles. Despite long-lasting increases in progesterone during gestation and an increase in estrogen before delivery, the uterus keeps a stable Per1::Luc rhythm throughout the pregnancy. The study suggests the importance of stable circadian environments for fetuses to achieve sound physiology and intrauterine development. This idea is also supported by epidemiological and animal studies, in which pregnant females exposed to repeated shifting of the LD cycles have increased rates of reproductive abnormalities and adverse pregnancy outcomes. Leading from this, we introduced artificial circadian environments with controlled lighting conditions to human preterm infants by developing and utilizing a specific light filter which takes advantage of the unique characteristics of infants’ developing visual photoreceptors. In spite of growing evidence of the physiological benefits of nighttime exposure to darkness for infant development, many Japanese Neonatal Intensive Care Units (NICUs) still prefer to maintain constant light in preparation for any possible emergencies concerning infants in incubators. To protect infants from the negative effects of constant light on their development in the NICU, we have developed a new device similar to a magic mirror, by which preterm infants can be shielded from exposure to their visible wavelengths of light even in the constant light conditions of the NICU while simultaneously allowing medical care staff to visually monitor preterm infants adequately. The device leads to significantly increased infant activity during daytime than during night time and better weight gains.

## What is the Physiological Significance of Circadian Rhythmicity in the Reproductive Organs of Pregnant Mothers?

**Figure 1:**
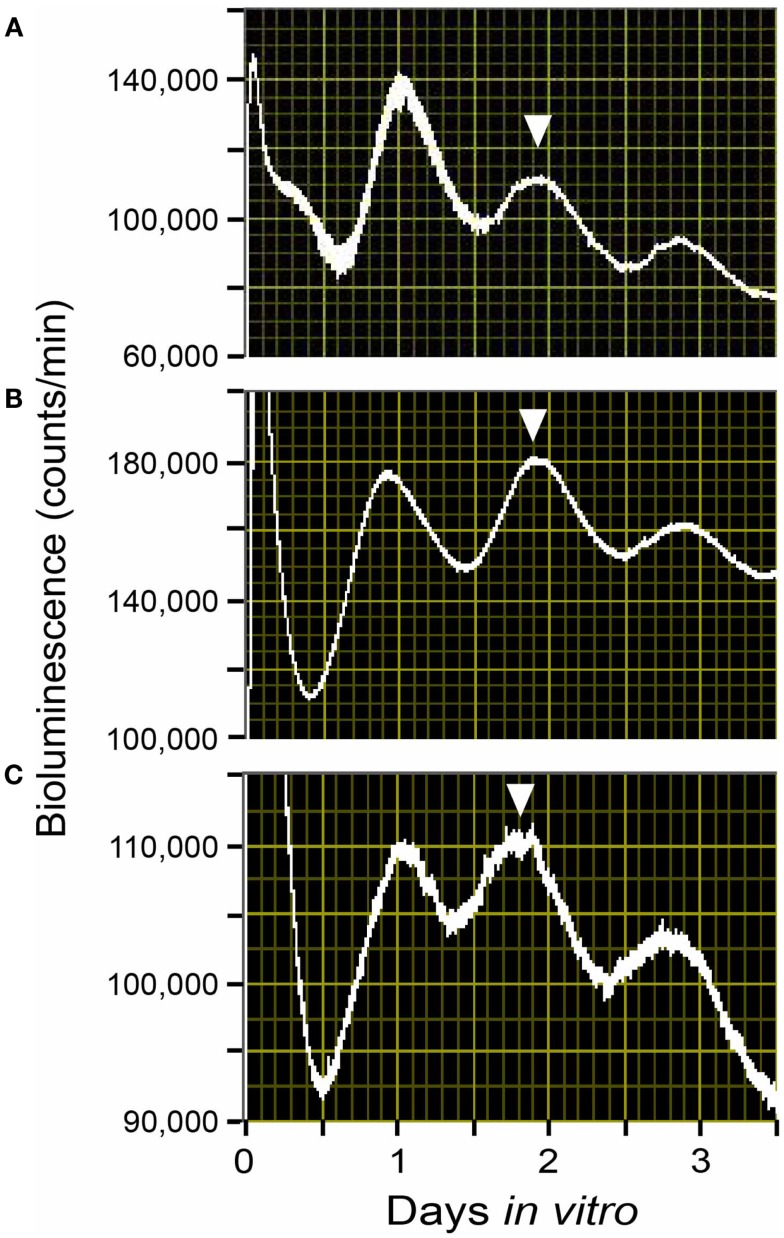
**Rhythms of Per1::Luc light emission by uterus explants from Per1::Luc rats in a light-dark cycle (LD)**. Shown are raw data from uterus tissues from ad lib-fed Per1::Luc female rats in non-pregnancy **(A)**, embryonic day 12 **(B)**, and embryonic day 22 **(C)** in a 12:12 h LD cycle. The phase of the tissue *in vivo* has been considered to be best reflected by the phase of the peak *in vitro* during the first full subjective day (1–2.5 days after explant) as previously described (9, 49). The phase of these peaks is consistent from animal to animal. Here, the phase statistically chosen is indicated by the inverted triangles.

Both epidemiological and animal studies have indicated that one of the physiological significances of circadian rhythmicity in the reproductive organs during pregnancy is the positive effects of circadian environments on proper fetal development (1). Using uterine explants from Per1::Luc rats and Per1 mRNA expressions by *in situ* hybridization, we have shown that the circadian property of the molecular clocks in the uterus and placenta is stably maintained from non-pregnancy, right through to the end stage of pregnancy under regular light-dark (LD) cycles. Despite long-lasting increases in progesterone during gestation and an increase in estrogen before delivery, the uterus and the decidua portions of the placenta keep a stable Per1 rhythm throughout the pregnancy (Figure 1). Our study’s findings suggest the importance of stable circadian environments for fetuses to achieve sound physiology and intrauterine development.

The concept of a stable circadian uterus environment having positive effects on fetal development is also supported by clinical studies in which pregnant females exposed to repeated shifting of their LD cycle had increased rates of reproductive abnormalities and adverse pregnancy outcomes such as preterm delivery and particularly low birth weight of offspring. Several epidemiological studies have identified associations between shift work or repeated travel across time zones and reduced fertility (2) as well as negative pregnancy outcomes, including increased incidence of low birth weight, preterm birth, and miscarriage (3–5). However, whether these adverse outcomes are due to circadian dysregulation or some other lifestyle factor associated with shift work has not been clearly determined.

A recent study using a rat model also reported that exposure of pregnant rats to constant light, which disrupts the circadian environment for fetuses, induced intrauterine growth retardation, and also lowered corticosterone production in fetal adrenal glands (6). Other studies using mice models indicated that repeated shifting of the LD cycle of pregnant mice increased rates of miscarriage and induced hyperleptinemia and hyperinsulinemia in offspring lasting into adulthood (7, 8). Shifts in rats’ LD cycle are known to transiently disrupt normal phase relationships between the SCN and peripheral oscillators (9), thus desynchronized circadian rhythms, either between central and peripheral maternal tissues or between maternal and fetal tissues (or both), may in part contribute to the adverse effects of chronic environmentally mediated circadian disruption on pregnancy outcomes.

## Preterm Infants Lose Circadian Information from the Mother

The preterm birth is a typical example in which all the circadian information from the mother to fetus is prematurely lost. The intrauterine environment is a rhythmic environment highly controlled by interactions between maternal-fetal hypothalamus, in particular the suprachiasmatic nuclei (SCN), and placenta using maternal hormonal cues (10, 11). Therefore, the development of preterms’ circadian rhythms may be hampered by environmental disturbances in postnatal periods when the intimate mother-fetus relationship is dramatically altered by premature birth. Furthermore, preterm infants are exposed to continuous or unpredictable light illumination and sounds for several weeks or months in the Neonatal Intensive Care Unit (NICU) and intermediate nursery (12). A multicentered study by the National Institute of Child and Human Development (NICHD) Neonatal Research Network indicated that 16% of extremely low birth weight infants are small for gestational age at birth, and that follow-up data at 18–22 months corrected age shows that 40% of the extremely low birth weight infants still have weights, lengths, and head circumferences less than the 10th percentile (13). Clearly, the NICU is not a surrogate for the maternal placental unit.

Maternal signals such as melatonin, corticosteroids, nutrients, body temperature and uterine mortality are suggested to mediate the effect of the LD cycle on the fetus. It has already been determined that the fetal brain clock, the SCN, already oscillates *in utero* (14, 15), and subsequent studies have focused on the interactions between maternal signals and fetal SCN to try to better understand fetal physiology. In the uterus, the fetal SCN do not have access to the outside world’s LD cycles and the neural connections to convey light information from the eyes to the fetal SCN are still under development, resulting in the developing fetus needing to rely on maternal rhythms (11). Each maternal signal most likely includes a level of redundancy, and may have different targets in the embryo/fetus during development. There is evidence for three maternal candidate signals acting during gestation on the entrainment of fetal rhythms: melatonin, maternal feeding time, and glucocorticoid.

Direct evidence for maternal melatonin as a possible entrainer (synchronizing factor) for the fetal SCN has been reported in a study using the capuchin monkey in constant light (16). Melatonin receptors are present in the capuchin monkey, human, and rodent fetal SCN (17, 18). Suppression of maternal melatonin secretion by exposing pregnant female capuchin monkeys to constant light during the last third of gestation, shifted Bmal1 and MT1 melatonin receptor expression in the fetal capuchin monkey SCN and this shift was reverted by daily melatonin replacement. These data suggest that maternal melatonin serves a role as an entrainer for the fetal primate SCN (16). This result was also indirectly supported by rodent studies, in which timed injections of melatonin into SCN-lesioned or pinealectomized pregnant mothers enabled them to give birth to pups with normal circadian rhythms of locomotor movements and water drinking (19, 20). Removal of the pineal glands, however, does not seem to disrupt maternal-fetal communication of circadian phase in the rat fetal clock, indicating that the rhythmic hormonal outputs from the pineal glands may not be the only maternal entrainer (21).

**Figure 2:**
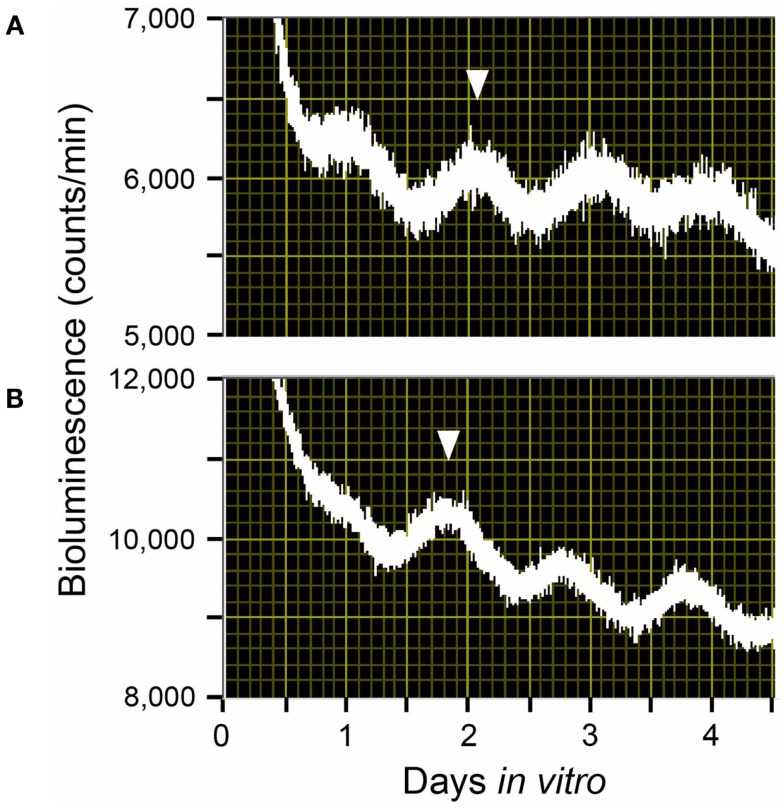
**Rhythms of light emission by fetal SCN explants**. Shown are raw data from **(A)** a fetus of an ad lib-fed control Per1::Luc pregnant rat and **(B)** a fetus of a Per1::Luc pregnant rat that had been exposed to a 4-h restricted-feeding (RF) regimen for 21 days after mating. The phase of these peaks is consistent from animal to animal. Here, the phase peak statistically chosen is indicated by the inverted triangles.

**Figure 3:**
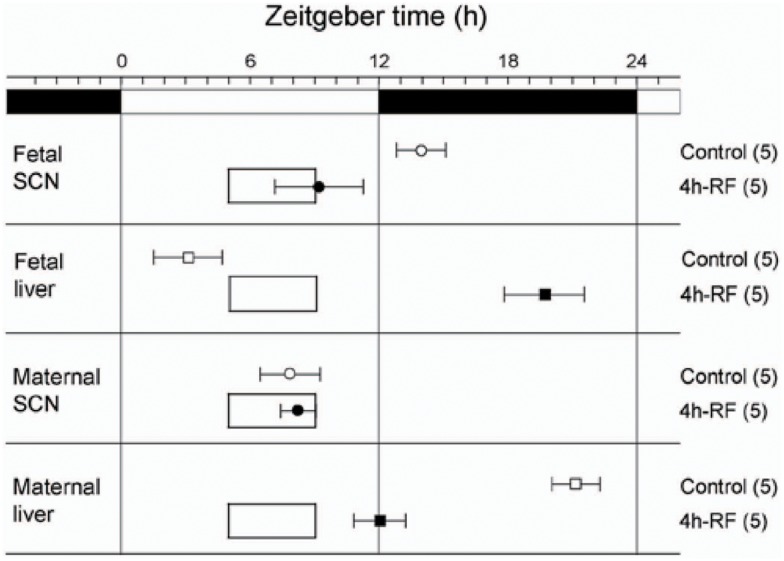
**Effects of 4 h restricted-feeding on tissue luciferase rhythmicity**. The average times (±SD, shown by error bars) of peaks from the different tissues are plotted against the LD cycle shown at the top of each panel. The timing and duration of food availability at ZT5-9 is indicated by open boxes in each section. The sample size is shown in parentheses. The phases of Per1::Luc fetal SCN, fetal liver, and maternal liver rhythmicity were significantly different from control values in all groups of RF rats (one-way ANOVA, *p* < 0.01); the phase of maternal SCN rhythmicity was not significantly different between control and 4h-RF groups.

Maternal feeding has also been reported to control fetal clocks in rodent models. With a Per1::Luc transgenic pregnant rat model under a 4-h restricted-feeding (RF) schedule, we found that maternal feeding entrains the fetal SCN and liver independently of both the maternal SCN and the LD cycle. In our study, maternal RF phase-advanced the fetal SCN and liver by 5 and 7 h respectively within the 22-day pregnancy (Figures 2 and 3) (22). In contrast, however, another recent study using a pregnant rat model under a 6-h RF schedule reported an effect from maternal feeding on the daily profiles of AVP and c-Fos mRNA in *in situ* hybridization of the SCN of 1-day-old pups in constant light, but no effect from maternal feeding when conducted in an LD cycle (23). Both studies indicate that maternal feeding affects fetal clocks. However, it is difficult to analyze the difference in results between the two studies for RF in LD cycles since the studies had different maternal feeding schedule, tissue sampling method, time resolution of sampling, and developmental stage of assessed brain tissues such as fetal or neonatal SCN. Unfortunately, to date, except for a few pilot studies, no systematic clinical studies on primates or human infants to examine the effects of maternal feeding on the fetus or preterm infants have been performed (24).

The other candidate maternal signal for controlling the fetal clock is glucocorticoid. Findings that corticosterone shifts the phase of clock genes in fibroblasts and liver tissue have created interest among researchers in examining the effects of glucocorticoid on the fetal clock (25–27). However, the effects of glucocorticoids on fetal SCN have yet to be reported in either animal or clinical studies. Further studies are required to understand how glucocoricoid affects fetal clocks during pregnancy.

## How Can We Make Artificial Circadian Environments for Preterm Infants?

The earlier-mentioned candidate maternal signals may be utilized for artificially generating circadian rhythmicity in preterm infants to improve postnatal development in incubators. There are, however, possible side effects in clinical applications of melatonin and glucocorticoids that we should be aware of.

### Melatonin

No multicentered trials have investigated melatonin’s effect on unborn fetal development. The National Institutes of Health (NIH) regards melatonin as “possibly unsafe” for use during pregnancy, also indicating that it may be dangerous for young children. In particular, the NIH suggests that melatonin may interfere with the development of child reproductive hormones (MedlinePlus, NIH web information). In a rat neonatal model, melatonin-induced inhibition of GnRH-stimulated calcium signaling has been reported to suppress gonadotropin release from neonatal gonadotrophs (28). A molecular study with an avian model has also indicated that melatonin induces the expression of gonadotropin-inhibitory hormone, suggesting that melatoin inhibits gonadotropin release (29). Additionally, melatonin helps to regulate female reproductive hormones such as estrogen, progesterone, and prolactin, and may affect females’ ability to maintain pregnancy (MedlinePlus, NIH web information). A possible side effect of induced seizure in neurologically disabled children has been also reported in melatonin administration, which was originally administered to treat sleep disturbances (30). In contrast, in a relatively small clinical trial, melatonin was given orally to newborn infants who had suffered birth asphyxia, and was shown to significantly reduce plasma levels of malondialdehyde and nitrate/nitrite, two robust indicators of oxidative stress, suggesting that melatonin contributed to reducing oxidative stress in neonates (31, 32). In a rat model, fetal clock was reported to be regulated by timed melatonin administration to pregnant rat mothers (33). Products made with melatonin are generally safe when used in healthy adults for short periods of time. However, melatonin’s role in pregnancy and human development is poorly understood and under-researched.

### Glucocorticoids

Fetuses at risk of premature delivery are now routinely exposed to maternal treatment with synthetic glucocorticoids. In randomized clinical trials, synthetic glucocorticoids substantially reduced acute neonatal systemic morbidity and mortality after premature birth and reduced intraventricular hemorrhage. However, there is now considerable experimental and clinical evidence that postnatal administration to preterm infants is consistently associated with impaired brain development. In rodents, glucocorticoids are associated with reduced neuron cell proliferation and a decrease in total cell numbers, leading to impaired brain growth, and development. Indeed, in rodents, exposure to glucocorticoids before or immediately after birth may be associated with marked upregulation of neuronal and progenitor cell apoptosis, particularly in the hippocampus where they deplete the pool of neural progenitor cells and reduce hippocampal growth (34). In a human study comparing 11 newborns exposed to postnatal dexamethasone to 30 unexposed newborns, those exposed had smaller overall brain volumes at term-equivalent age, including cerebellar volume (35). A recent study also reported that postnatal exposure to clinically routine doses of hydrocortisone or dexamethasone was associated with impaired cerebellar, but not cerebral, growth (36). In a randomized controlled trial of early postnatal dexamethasone therapy, those exposed to dexamethasone were found to have decreased height and head circumference, and worse outcomes on motor and cognitive tests carried out until the subjects were 8 years of age (37). On the basis of these data, the American Academy of Pediatrics recommends against the use of high-dose dexamethasone (38).

As a result of the above-mentioned possible side effects, the use of melatonin and glucocorticoid to entrain the fetal clocks during pregnancy may not be appropriate. However, regular cycles of maternal feeding, which can be naturally achieved by pregnant mothers as part of their daily lifestyle, can be safely applied for both fetal clock entrainment and proper fetal development.

## Lighting may be the Smartest Way of Substituting Maternal Signals to Entrain Biological Clocks of Preterm Infants

Although the exact maternal signals actually employed in maternal-fetal clock synchronization have not been clearly identified, basic and clinical studies have suggested an alternative way of entraining the developing clocks of preterm infants by using a signal which is quite different from the earlier-mentioned candidate maternal signals – that is, with a daily LD cycle. Fortunately, unlike melatonin and cortisol, lighting has been demonstrated to have no adverse side effects by a multicenter trial in the US. In the 1980’s, Glass et al. (39) raised the concern that continuous light exposure in NICUs may deteriorate retinal development of preterm infants and increase the incidence and severity of retinopathy of prematurity (ROP). The trial, however, showed no change in the incidence of ROP, blindness, or other major visual defects (40).

Human preterm infants are able to detect light before term – a much earlier developmental stage than other mammals. Mouse melanopsin starts functioning on day of birth and mouse rhodopsin and cone opsins start functioning 2 weeks after birth (41). In contrast, Robinson and Fielder (42) reported that preterm infants can respond to light from 30 weeks of conceptional age, based on measurements they had made of the pupillary light reflex (PLR). Another study by Hao and Rivkees (43) using a baboon model indicated that by as early as 28 weeks, the circadian system of human preterm infants might be biologically responsive to the effects of light. Therefore, we might be able to entrain the circadian clocks of preterm infants simply by using light.

In clinical studies, Rivkees et al. (44) reported that exposure of premature infants to a regular LD cycle in the hospital nursery induces distinct patterns of rest-activity that are apparent within 1 week after discharge, suggesting that the circadian clock of developing infants is entrained by lighting conditions. Kennaway et al. (45) also found that the delayed development of the melatonin rhythm in some preterm infants could be advanced by regular LD cycles. A regular LD cycle was also reported to improve body growth of preterm infants during hospitalization in the NICU (46). Mann et al. (47) reported increased sleep and growth in preterm infants subjected to a regular LD cycle in the nursery but this finding was not evident until 6 weeks post discharge. Previous studies also found that by 4 months of age, there was a significant increase in body weight, head circumference, and lengths of infants discharged from a LD cycle exposure room compared with a dim room (48).

In addition to being able to entrain the developing clocks of preterm infants from scratch, a regular LD cycle is also an effective method to reset a biological clock disrupted by constant light – a lighting condition which is still standard in many NICUs. In animal studies, a transgenic neonatal mouse model has shown that continuous lighting conditions disorganize harmonized cellular synchrony in the SCN, the neonatal brain clock, and that regular LD cycles reorganized the neonatal clock disrupted under constant light to have a regular circadian rhythm (49, 50). It has also been reported that the characteristics of the perinatal photoperiod have lasting effects on the circadian rhythms expressed by the SCN neurons as well as on behavior, and set the responsiveness of the biological clock to subsequent changes in photoperiod. In addition, it was stated that perinatal exposure to winter-like seasonal light cycles, which consist of 8 h light periods and 16 h dark periods, induce persistent elevated depressive and anxiety-like behaviors in rodents (51). Interestingly, light perceived by melanopsin-expressing retinal ganglion cells were recently reported to influence mood and learning behavior in a mouse model (52).

As described in the next section, our group also investigated the development of the light detection systems of preterm infants, focusing particularly on melanopsin and rhodopsin, photoreceptors in the retina.

## How Do Preterm Infants Perceive Light-Dark Cycles to Adapt Themselves to Lighting Environments?

The human retina has three types of visual sensors (photoreceptors): rhodopsin, cone opsins, and melanopsin. Previous studies have demonstrated that, in humans, rhodopsin begins to function a few days after birth (53) and cone opsins start functioning 1 month after birth (54). KO mouse studies revealed that melanospin starts to function on day of birth, earlier than the other photoreceptors while rhodopsin or/and cone opsins seems to suddenly start working approximately 2 weeks after birth (41). These studies suggest that during development, photoreceptors start to function in the order of melanospin, rhodopsin, and cone opsins. Mouse studies also proved that melanopsin, in addition to rhodopsin and cone opsins, is able to control the PLR (55). We recently reported a preterm infant of 33 weeks’ gestational age who did not show a PLR to a 600 nm-wavelength monochromatic light, which is outside the range of perceptibility of melanopisn but within the range of perceptibility of the other photoreceptors (56). The case report suggested the possibility that only melanopsin is able to function within the early stages of development of human fetuses and preterm infants. In this study, we examined the hypothesis that only melanopsin contributes to the visual perception of immature preterm infants and that other photoreceptors only develop well enough to function later in life.

To investigate the hypothesis, preterm infants of gestational age between 31 and 36 weeks were selected for observation (57). The subjects were divided by age into either a 31–33 week gestational age group or a 34 to 36-week gestational age group (*n* = 5 for each group). All protocols were conducted in compliance with Tohoku University’s Human Subjects Committee Institutional Review Board. The subjects underwent pupil diameter measurements under different levels of illumination of either 15.3 μW/cm^2^ of white light or 7.57 μW/cm^2^ of a monochromatic light of 600 nm (half bandwidth<5 nm), both of which were generated by a 10 W EN20-1 bulb (Heine, Hersching, Germany) with or without a monochromatic light filter (Ashahi-bunkoh, Tokyo, Japan). The irises of each subject were videotaped for 1 min in the dark prior to light exposure, and for 5 s during light exposure. The PLR was examined during the dark period of the neonatal ward between 20:00 (1 h after the onset of dim light,<5 lux) and 21:00 h.

Images videotaped during the sessions were digitized and pupil sizes were measured. After the procedures were completed, a video-editing program (Adobe Premiere 6.0) was used to capture a set of two still video images (the first, an image of the eye before light exposure, and the second, an image of the eye during light exposure) from each subject representing each experimental condition. The iris diameter for each session was taken with Image J, NIH image software (NIH, MD, USA).

**Figure 4:**
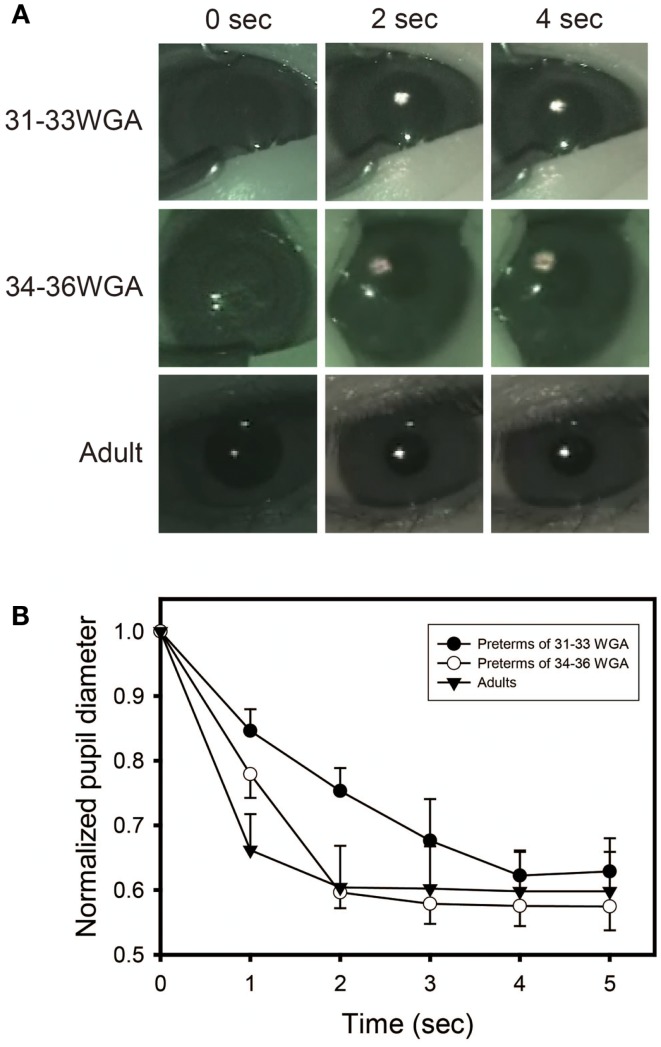
**Preterm infants exhibit a slower pupillary light reflex (PLR) to white light**. **(A)** Preterm infants and adults all showed consensual pupillary constriction in response to 5 s of white light (quartz halogen source, 15.3 μW/cm^2^), but the constriction was slower in preterm infants than in adults. Preterm infants of 31–33 weeks’ gestational age exhibit a slower PLR to white light, particularly observed during the first 2 s after light exposure. **(B)** Time courses of PLRs in preterm infants and adults to white light (quartz halogen source, 15.3 μW/cm^2^, 5 s) The diameter of the normalized pupil is expressed as a ratio of its size to the size of the dark-adapted pupil diameter (recorded just before light exposure, time 0 on the graph). The normalized pupil diameter “1.0” indicates the maximum diameter of the dark-adapted pupil. Values are mean ± SD for the five subjects per each group.

Figure 4 shows the puplillary constrictions after white light exposure for preterm infants and adults. When tested with white light, preterm infants of gestational ages of both 31–33 and 34–36 weeks and adults showed the same degree of high-amplitude constriction at 4 s after light exposure, confirming the infants as mature in sensitivity to light at both developmental stages as adults. The response speed of the PLR, however, was different between the three groups. Both infants of 34–36 weeks’ gestational age and adults showed a maximum pupillary constriction at 2 s after light exposure. In contrast, the preterm infants of 31–33 weeks’ gestational age exhibited a significant delay (two-way ANOVA followed by Bonferroni, two-tailed, *p* = 0.00 for both the preterm infants of 34–36 weeks’ gestational age and adults) and reached a maximum pupillary constriction at 4 s after light exposure. This is consistent with a previous report on the properties of the PLR in rodless coneless (rd/rd cl) mice, in which only melanopsin functioned but rhodopsin and cone opsins did not (58).

**Figure 5:**
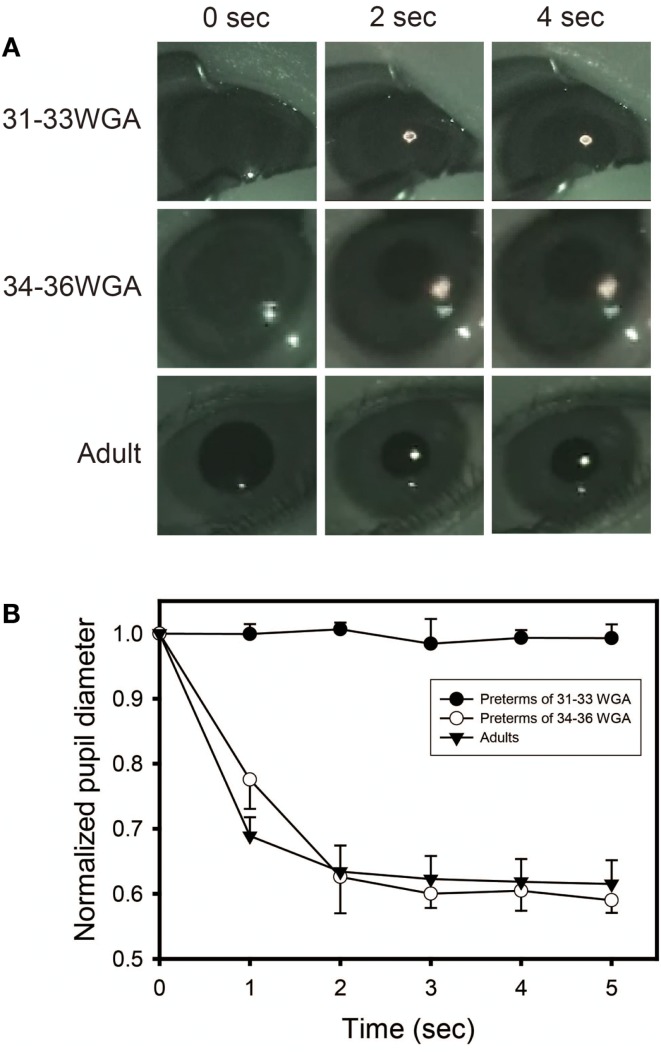
**Preterm infants of 31–33 weeks’ gestational age exhibit no pupillary light reflex (PLR) to 600 nm-monochromatic light**. **(A)** Pupillary constrictions in response to 5 s of 600 nm-monochromatic light (quartz halogen source, 7.57 μW/cm^2^) in preterm infants and adults were examined. Preterm infants of 31–33 weeks’ gestational age exhibit no PLR to 600 nm-monochromatic light while preterm infants of 34–36 weeks’ gestational age and adults showed PLRs. **(B)** Time courses of PLRs in preterm infants and adults to 600 nm-monochromatic light (quartz halogen source, 7.57 μW/cm^2^, 5 s). For details, see the legend ofFigure 4.

Next, to test more specifically whether only melanospin mediates the PLR at 31–33 weeks’ gestational age, we exposed preterm infants of gestational ages of both 31–33 and 34–36 weeks to 600 nm-wavelength monochromatic light, which is outside of the range of perceptibility of melanopsin but within the range of perceptibility rhodopsin and cone opsins. No sensitivity to a 600 nm-wavelength monochromatic light was observed in the preterm infants of 31–33 weeks’ gestational age (Figure 5). However, the same maximum pupillary constriction to a 600 nm monochromatic light as adults was observed once infants were at 34–36 weeks’ gestational age (two-way ANOVA followed by Bonferroni, two-tailed, *p* = 1.000). This indicates that rhodopisn, which can detect light of wavelengths between 600 and 610 nm, did not function in the infants at 31–33 weeks’ gestational age but did function at 34–36 weeks’ gestational age, suggesting that the rhodophsin of these infants starts to develop after melanopsin before term.

The data reveal for the first time that melanopsin may be the only photoreceptor working at the early stage of human development of 31–33 weeks’ gestational age, indicating the dominant role of melanopsin in preterms’ visual function. In addition, unlike our hypothesis or what has been stated in previous reports, this study showed that rhodopsin is likely to already start functioning before term at 34–36 weeks’ gestational age. Our data taken together with previous findings have widespread implications for the understanding of the functional development of human vision in preterm infants and suggest a possible scenario: that only melanopsin works for visual perception at the early stages of human development – at 31–33 weeks’ gestational age – and contributes to the detection of changes in environmental light radiance, rhodopsin starts to function sometime between 34 and 36 weeks’ gestational age and acts for image detection, and finally, cone opsins begin to function 1 month after birth (53, 54) and perform image and color detection. With a simple observation of the PLR, our study reexamined the possibility that human melanopsin and rhodopsin start functioning before term, that is, earlier than cognitive psychology has previously reported.

## Lighting Environments in NICUs – US and Japanese Studies

In clinical medicine, the importance of circadian biology is becoming increasingly appreciated as previously described. Yet little consideration has been given to the importance of regular LD cycles in the care of premature infants who are reared for extended periods in hospital nurseries. The issue of the most appropriate lighting conditions for NICUs is currently being debated in the US and Japan.

The practice of nursery lighting has also changed in the past several decades (44). In the US, a regular LD cycle was often used in hospital nurseries in the 1950s and 1960s, followed by continuous lighting when isolettes and NICUs were introduced. In contrast, continuous dim light was introduced in the 1980s and 1990s. Since the 1990s, in many NICUs in the US, Canada, and Europe, neither premature nor term infants are exposed to regular LD cycles. Rather, infants are continuously exposed to dim lighting, which is achieved by using low-level room lighting and crib covers. Dim lighting conditions have been supported by a Neonatal Individualized Developmental Care Assessment Program (NIDCAP), following the belief that infants should be dark-reared to mimic the lighting conditions *in utero* (10).

**Figure 6:**
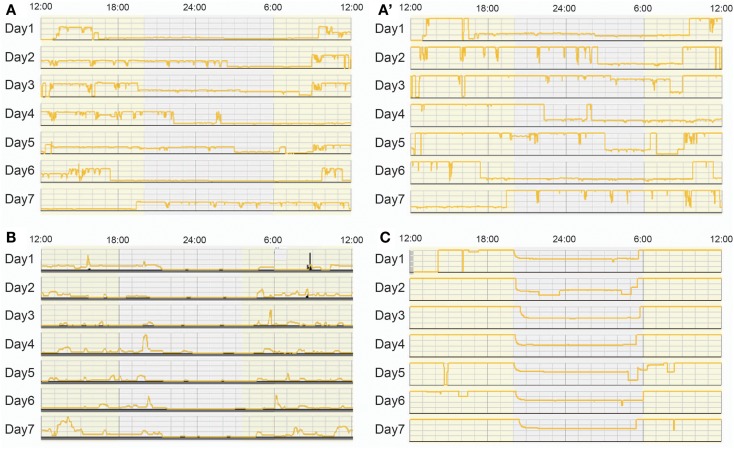
**Bed-site illumination in NICUs during a 7-day study**. Each plot represents light intensities inside an incubator in which measurements were continuously obtained at 1 min intervals for seven consecutive days. **(A)** Bed-site illumination in an NICU at a maximum illumination of 354 lux. **(A’)** The scale of the y axis of the same data as **(A)** is adjusted to 100 lux. A random light-dark schedule was observed and the illumination was over 25 lux most of the time throughout the 1-week observation. **(B)** Bed-site illumination in another NICU at a maximum illumination of 100 lux. The illumination was less than 25 lux most of the time throughout the 1-week observation (a continuous dark condition). **(C)** Bed-site illumination in another NICU at a maximum illumination of 100 lux. The illumination was more than 50 lux most of the time throughout the 1-week observation (a constant light condition).

At present, we are unaware of systematic surveys that have assessed nursery lighting conditions except for one study at Stanford University. The study recorded bed-site illumination over 5 days in an NICU and described that the NICU had rather irregular LD conditions, and that different parts of the nursery had different light intensity from day to day (12). Our study group organized a nationwide survey to assess the lighting conditions in 10 NICUs in Japan using actiwatches, automated illumination measurement, and recording devices, set inside incubators located at positions that were exposed to the average light intensity in each NICU, for seven consecutive days (59). Our data revealed that NICUs in Japan display a wide variety of lighting conditions from continuously dim, to continuously bright or regular LD cycled lighting conditions at different light intensities with different time schedules. The survey also showed that daily schedules of lighting conditions are strongly influenced by medical activities such as urgent hospital admissions of out born preterm infants (Figure 6).

Although a regular LD cycle has been recommended by circadian studies and the Guidelines for Perinatal Care by the American Academy of Pediatrics and the American College of Obstetricians and Gynecologists (60), debate concerning appropriate lighting conditions in NICUs in academic and clinical fields as well as the chaotic working situation inherent to emergency wards seem to prevent NICUs from giving preterm infants circadian information with regular LD cycles.

## What are the Effects of Circadian Environments on Development of Preterm Infants? – Japanese Study

**Figure 7:**
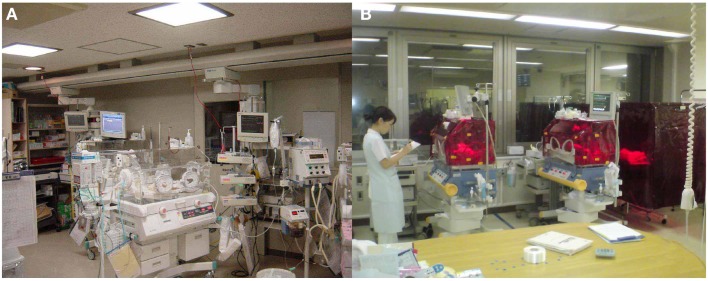
**Bed-site illumination before and after introduction of a monochromatic light red filter**. **(A)** In a typical NICU, continuous lighting was selected to allow instant reaction to emergency situations such as respiratory and cardiovascular dysfunctions, brain hemorriage, and infections. The background lighting was generally between 50 and 600 lux. **(B)** Artificial night in the experimental group was achieved by covering the isolettes or cribs with a light filter from 20:00 to 5:30. From 5:30 to 20:00, the light filter was removed from the isolette.

**Figure 8:**
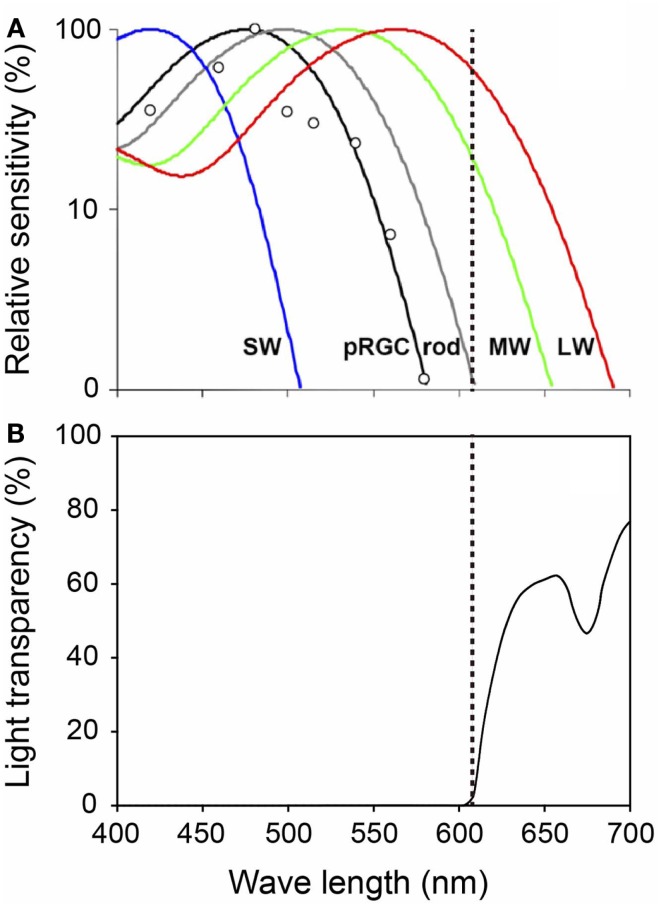
**Spectral properties of human photoreceptors and spectral transmission characteristics of the red filter**. **(A)** Spectral sensitivity of rod, cones, and melanopsin measured by pupil responses to light. Maximum light sensitivity wavelengths of human rods (R), S cones (SW), M cones (MW), and L cones (LW) are approximately 500, 420, 530, and 560 nm, respectively. The melanopsin (pRGCs) exhibit their peak sensitivity at around 480 nm. The dashed line on the figure indicates 610 nm. Figure cited by Zaidi et al. (61). White circles indicate the pupil responses to light in a blind woman. **(B)** Spectral transmission characteristics of the red filter. Note that the red filter cut light of wave lengths<610 nm perceived by melanopsin and rods, which function in preterm infants, while allowing wave lengths>610 nm perceived by adult MW and LW to pass through.

To gain some insight into a possible proper lighting environment, we examined the effect of a regular LD cycle on premature infants using a light filter which specifically cuts the wavelength of light that the melanopsin and rhodopsin of preterm infants detect. Artificial nights are introduced to the infants in the NICU by covering them with this red filter. Despite being covered, we can see the preterms through the filter and respond to any arising emergency as usual (Figures 7 and 8) (59). Premature infants who were born at birth weights of equal to or greater than 1000 g and less than 1500 g were studied. Infants were enrolled after achieving a medically stable condition between 30 and 34 weeks’ GA, which is approximately 4–8 weeks before anticipated discharge. Enrollment criteria included no significant eye disease (ROP grades 3 or 4), no major neurologic disorders (including intraventricular hemorrhage grades 3 or 4 or periventricular leukomalacia) and no major malformation syndromes. Infants were randomly assigned to either continuous lighting conditions (>30 lux; *n* = 20) or LD cycles (>30 lux, 5:30 am to 8:00 pm;<30 lux, 8:00 pm to 5:30 am; *n* = 19) using a computerized randomization system (Mebix, Tokyo, Japan). Infant activity and background lighting in the nursery rooms were continuously monitored for 24 h once every week from enrollment until discharge at approximately 38 weeks’ GA using a pair of Actiwatches (Minimitter Co, OR, USA), one for activity monitoring and another for illumination monitoring.

To assess whether there was diurnal variation in movement, day-night activity ratios were calculated. The amount of movements during the daytime period of 5:30 am to 8:00 pm was simply divided by the amount of movements for the nighttime period between 8:00 pm and 5:30 to calculate the day-night activity ratios. Then, to evaluate the effect of the filter on infants, the day-night activity ratios were compared between the group exposed to continuous lighting conditions and the group exposed to LD cycles using a Student’s *t*-test.

**Figure 9:**
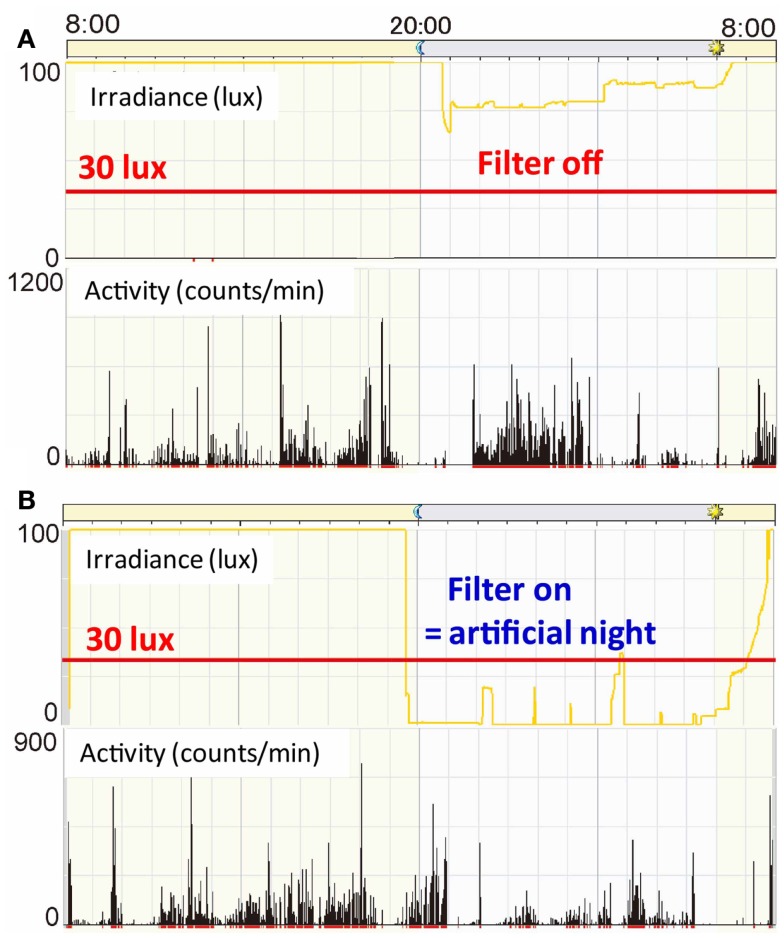
**Illumination inside incubators and actograms of rest-activity in representative infants who were with/without filters at 35 weeks gestational age**. Dark bars represent activity. The time of day is shown at the top. Note that circadian patterns of rest and activity are more apparent in infants with a filter at nights **(B)** than in infants with no filter**(A)**.

**Figure 10:**
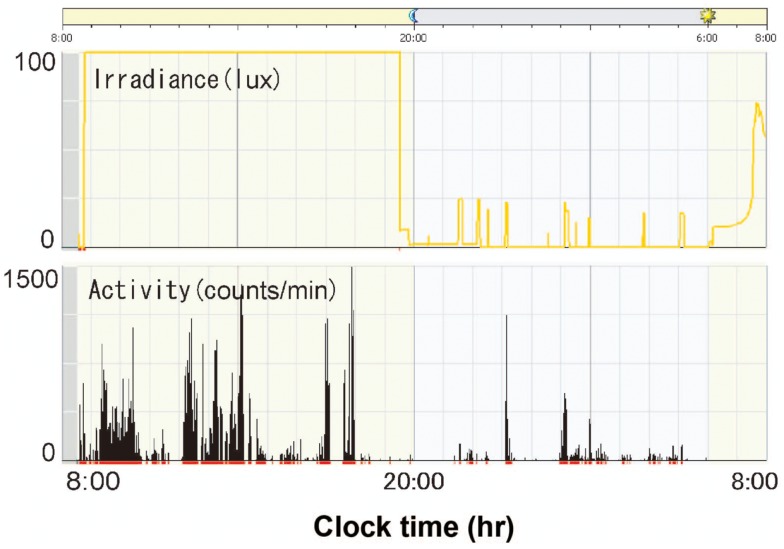
**Illumination inside incubators and actograms of rest-activity in a representative infant who were with a filter at 40 weeks gestational age**. Dark bars represent activity. The time of day is shown at the top. Note that distinct patterns of rest and activity are more apparent in infants of 40 weeks gestational age than infants of 35 weeks gestational age.

Figure 9A is a representative data from a preterm infant of 35 weeks gestational age exposed to fluorescent lights without a filter (59). The upper panel indicates 24 h levels of light intensity, which shows the NICU incubator always maintains a minimum level of 75 lux, which preterm infants can detect. The lower panel indicates 24 h activity levels of the preterm infant, which shows almost continuous movements during the 24-h. An Actiwatch counted 2927 movements/hour during daytime and 3991 movements/hour for nighttime, that is, more activity during nighttime. Figure 9B is another representative data from a preterm infant of 35 weeks gestational age exposed to artificial nights with a filter. The upper panel shows that the interior of the NICU incubator became completely dark (to the wavelength of light that preterm infants can detect) or sometimes just under 30 lux at most. The lower panel shows relatively more movements during daytime. Figure 10 is a representative data from a preterm infant of 40 weeks gestational age treated with the filter. After spending 9 weeks with artificial nights from the filter, the infant developed clear day-active patterns in his activity (59).

**Figure 11:**
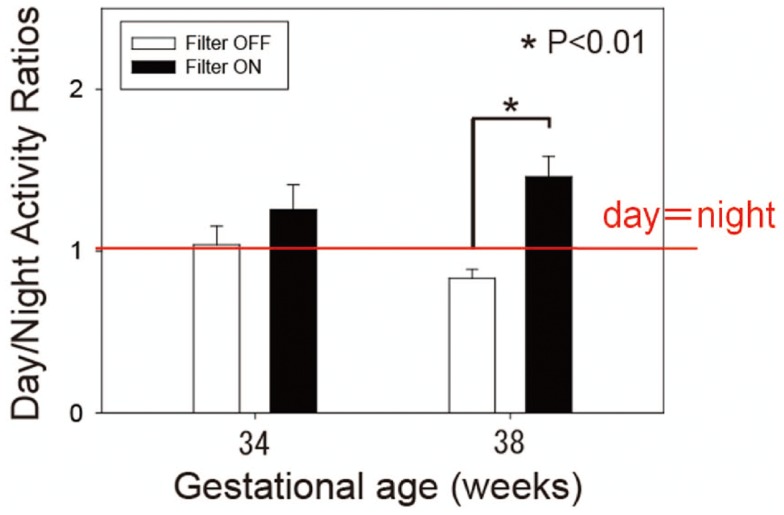
**Day-night activity ratios of preterm infants of 34 and 38 weeks’ gestational age**. White bars represent control infants without filter. Dark bars represent experimental group infants with a filter. Observe that filter cover at nights results in more daytime than nighttime activity at 38 weeks gestational age, but not at 34 weeks gestational age. Data from 19 control and 20 experimental group infants. *p < 0.01 by*t*-test.

Figure 11 is the summary data of day/night activity ratios at two developmental stages. At 38 weeks gestational age, preterm infants with the filter were more active during the day than at night (day-night ratio: 1.46 ± 0.15 SE) and *t*-test revealed a statistical difference between the two groups (59).

**Figure 12:**
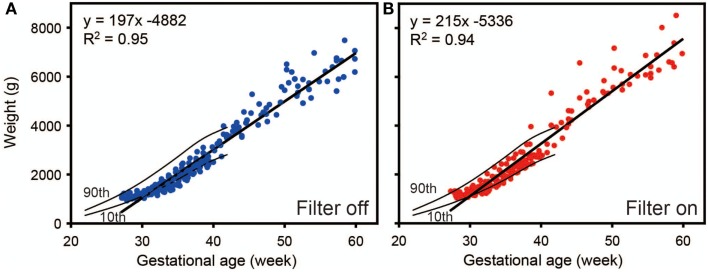
**Regression analysis of weight gain of preterm infants as a function of time**. Weight gain rate was decreased with **(A)** filter off compared to with **(B)** filter on. Results are from a generalized estimated equation for repeated measures. Data from 20 control and 19 experimental group infants. Tenth and ninetieth centile curves of birth weights from 22 to 42 weeks’ gestational age were also drawn in the figures (62).

In the same study groups, we compared the weight gain between the infants kept under filters and those not kept under filters. As we can see in Figure 12, the longitudinal distribution of body weights goes down in preterm infants without the filter (ANCOVA, *p* < 0.05), indicating that preterm infants exposed to regular LD cycles with the filter had more weight gains compared to preterm infant without the filter by 60 weeks of corrected gestational age (59). Like mentioned in previous reports (46, 47), infants in LD cycles had more weight gain than those in constant light.

## Near Future Goals

The biological clock has been reported to be the only physiological system which can collect light information from photoreceptors in the retina and then convey that information to organs within the whole body through hormonal signals and neural transmission. Ascertaining the details of “the light biological circuit” and determining the most appropriate lighting environments for organisms might be the most appropriate course for achieving an effective way to obtain proper development of preterm infants. In the near future, we might be able to realize better development of preterm infants by switching on and off “the light biological circuit” by exposing them to specific wavelengths of light within carefully designed LD cycles.

## Conflict of Interest Statement

The authors declare that the research was conducted in the absence of any commercial or financial relationships that could be construed as a potential conflict of interest.
